# Supplementary material for: The A-B transition in superfluid helium-3 under confinement in a thin slab geometry
Source: Nat Commun. 2017 Jul 3;8:15963. doi: 10.1038/ncomms15963 (PMC5500879; doi:10.1038/ncomms15963)
Supplement: Supplementary Information [file ncomms15963-s1.pdf]

Title of file for HTML: Supplementary Information
Description: Supplementary Figures, Supplementary Notes and Supplementary References

Title of file for HTML: Peer Review File
Description:

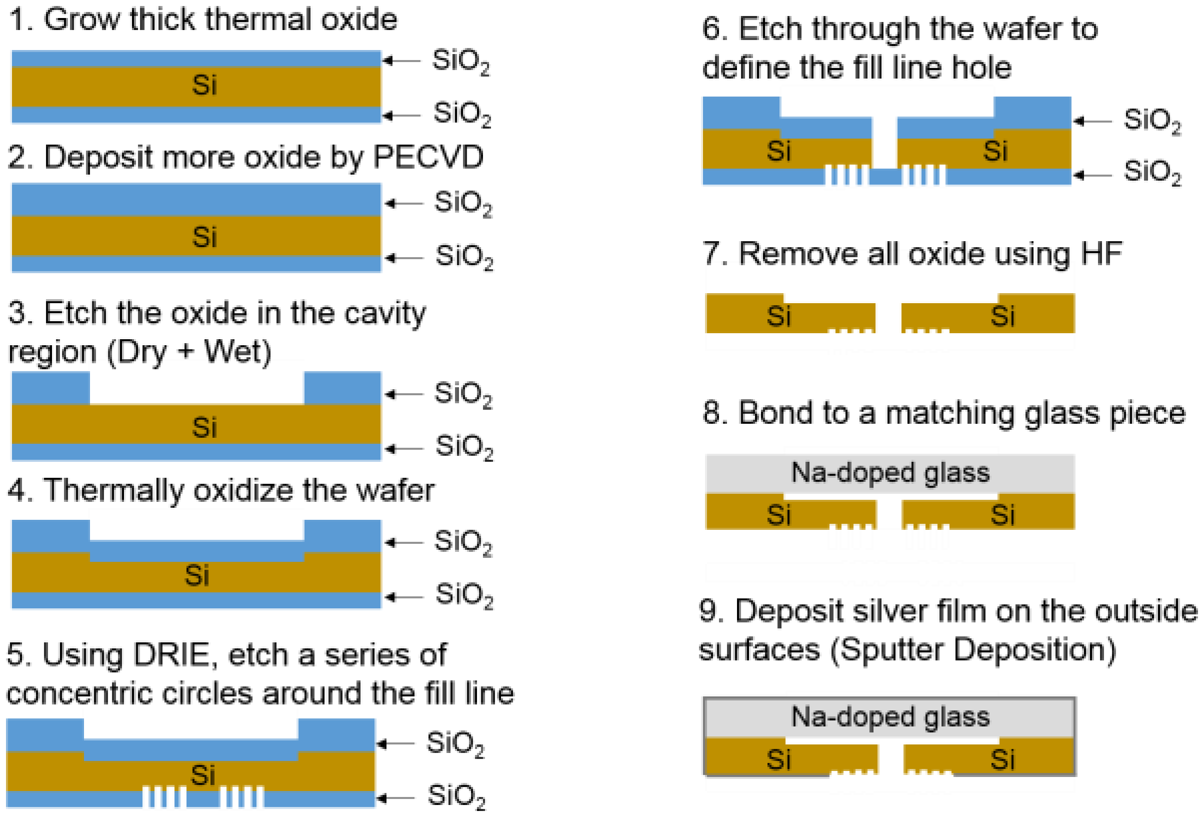

Supplementary Figure 1: Depiction of Silicon Fabrication Process

Process flow for patterning of Silicon wafer. 1. Growth of thick oxide – using a wet oxidation process in an atmospheric pressure tube furnace. 2. Deposition of secondary oxide layer, followed by patterning, exposure and 3. dry/wet etch to strip oxide. 4. Further thermal oxidation to define cavity in silicon. 5. Pattern backside to define concentric circles for fill line attachment. 6. Reactive ion-etch to define fill line. 7. Strip off all oxide, clean and characterize surface 8. Anodic bonding step to complete cavity. 9. Deposit silver film by sputtering.

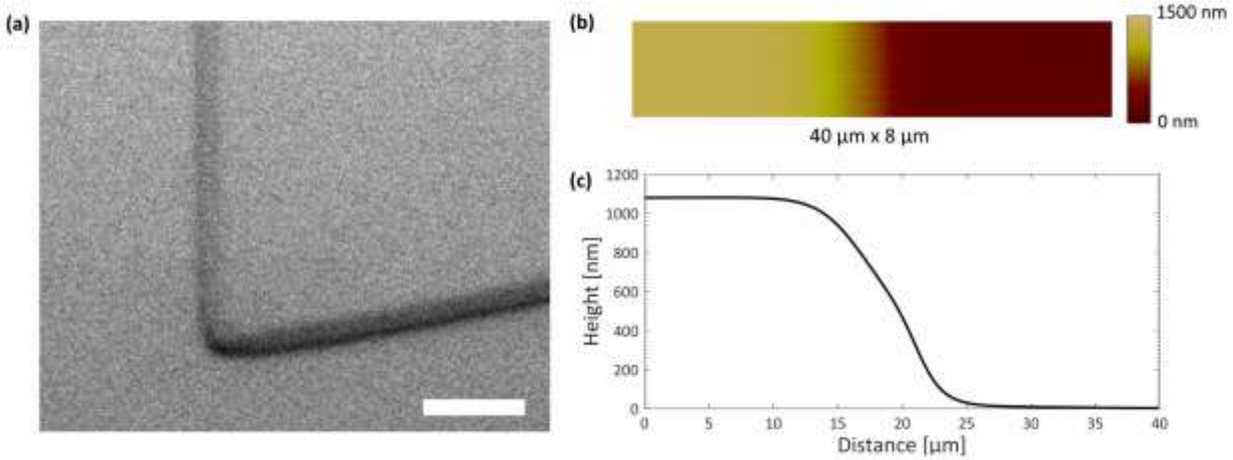

Supplementary Figure 2: Detail at Cavity Edge

(a) Scanning electron micrograph showing the inner cavity wall and the edge of the central channel (the scale bar is 40 µm). The walls of the cavity are rounded due to the oxidation process with a lateral extent of approximately 10 µm. The A phase is stabilized well below the studied A-B transition in this region near the cavity boundary, since the cavity depth is less than 1.08 µm there.

(b) Atomic force microscope image of step at the outside cavity wall. Scanned region is 40 µm × 8 µm.

(c) Line slice through the AFM scan showing clearly the profile of the cavity wall. Evident from this plot is the size of cavity of 1.08 µm.

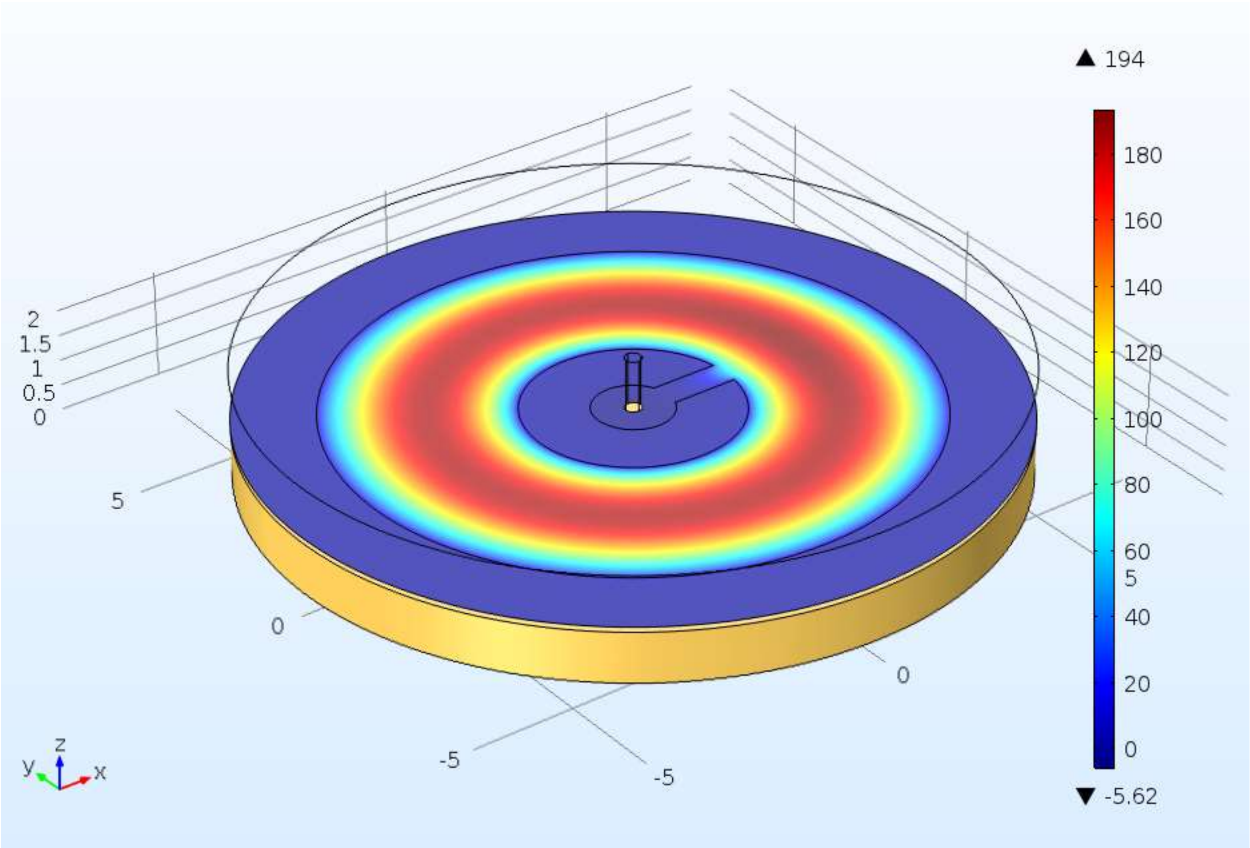

Supplementary Figure 3: Depiction of deflection of cell walls at 5.6 bar

Here we show the deflection of the cell walls (in nm) at 5.6 bar computed using COMSOL. The central "hub" region has a minimal deflection and is expected to be in the A-phase when the annular region is in the B phase.

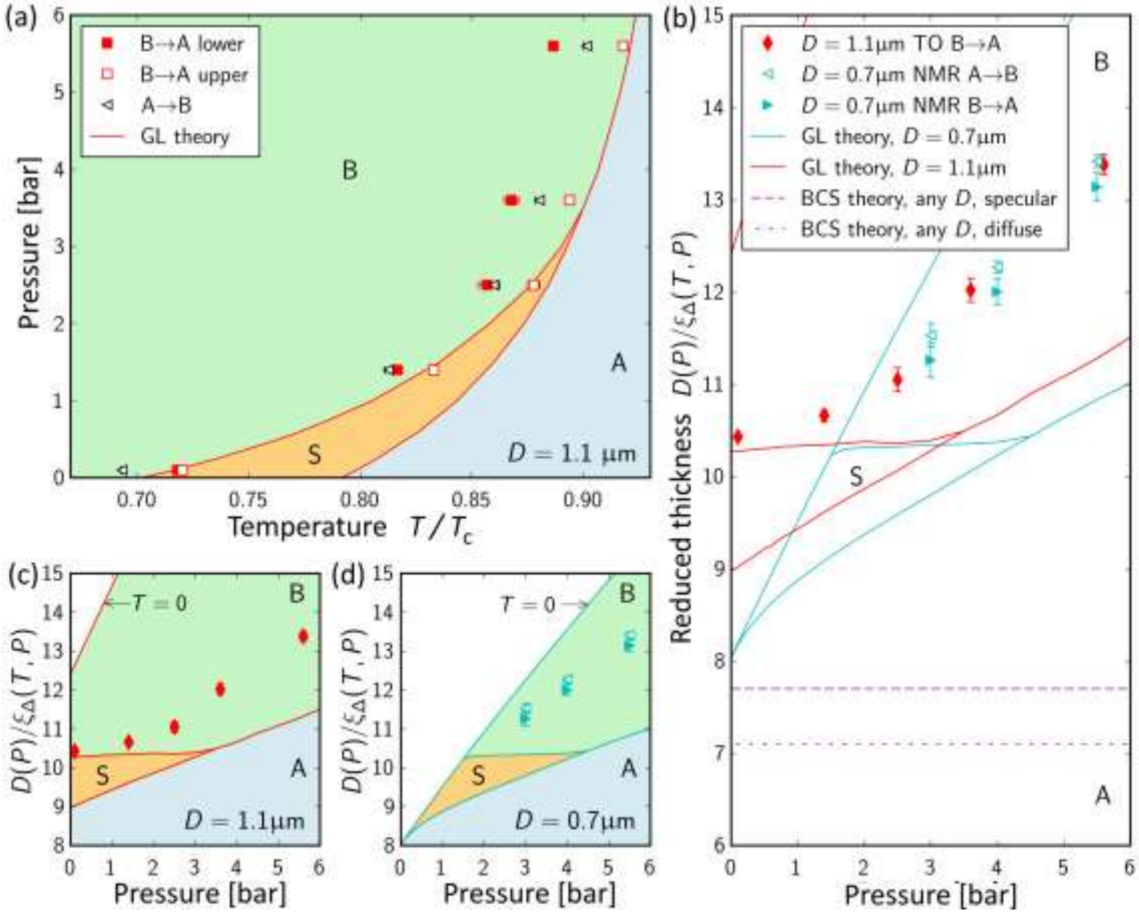

Supplementary Figure 4: Phase diagram of superfluid helium-3 confined in slab geometry.

(a) The A-B transition in the D=1.1 µm slab (this experiment) in the temperature-pressure plane. The start, $T_{\mathrm{BA}}^{\mathrm{lower}}$ (filled squares), and completion, $T_{\mathrm{BA}}^{\mathrm{upper}}$ (open squares), of the B to A transition on warming correspond to the equilibrium B-A transition in the thinnest (fixed D=1.08 µm near the edge) and thickest (pressure-dependent D) parts of the cavity respectively. On cooling the A phase supercools down to T_{AB} (black triangles). Solid red lines and blue/orange/green areas show the A/stripe-S/B phase diagram predicted by the Ginzburg-Landau (GL) theory[1] which are derived using the strong coupling parameters calculated by Serene and Sauls,[2] to be compared to $T_{\mathrm{BA}}^{\mathrm{lower}}$. The A phase is observed in the predicted region of stability of the stripe phase (orange), demonstrating the inaccuracy of the strong coupling parameters used for the calculation. (b) The reduced thickness D/ξ_Δ representation of the phase diagram allows to compare this torsion oscillator (TO) experiment (in this representation $T_{\mathrm{BA}}^{\mathrm{lower}}$ and $T_{\mathrm{BA}}^{\mathrm{upper}}$ coincide) with the NMR experiment on a D=0.7 µm slab.[3] The two datasets' collapse demonstrates the universality of D/ξ_Δ at the A-B transition. These measurements strongly deviate from the prediction of the weak coupling (BCS) theory, shown here for diffusely and specularly scattering cavity walls. The phase boundaries derived within the GL theory for D=1.08 µm and 0.7 µm (red and cyan

lines; also shown separately in (c) D=1.08 μm and, (d) D= 0.7 μm) alongside relevant TO (c) and NMR (d) experimental results. The disagreement between the experiments and GL theory emphasizes the current limited understanding of the strong coupling parameters at low pressure that leaves the stability of the stripe phase uncertain. The sensitivity of the calculated phase diagram to details of strong coupling can be gauged by comparing this figure to Fig. 6 in the main text. Error bars represent standard errors of the mean.

Supplementary Note 1: Calculating A-B transition properties and fits near T_{BA}.

Here we describe the procedure to obtain the values presented in Table 1 of the main text and to the superfluid fraction data while warming through T_{BA}. To fit for the best value of $D/\xi_\Delta\,(P, T_{\mathrm{BA}}/T_{\mathrm{c}})$ at a given pressure we followed the procedure outlined below. We divide the annular channel into N equal radial elements.

1. We obtain linear fits for $\rho_{\mathrm{s}}^{\mathrm{B}}(T/T_{\mathrm{c}})$ and $\rho_{\mathrm{s}}^{\mathrm{A}}(T/T_{\mathrm{c}})$ near $T_{\mathrm{BA}}/T_{\mathrm{c}}$.
2. From the finite element modeling, we obtain a table of $D(r, P)$ where r is the radius of a fluid element measured from the TO axis at any given pressure, P using a value of 31 nm.bar^{-1} for the maximum deflection.
3. We write the superfluid fraction

$$\frac{\rho_{\mathrm{s}}^{\mathrm{Fit}}}{\rho} = \frac{\sum_{i=1}^{N} \frac{\rho_{\mathrm{s}}^{\mathrm{A\,(or)B}}}{\rho}\left({}^{T}/{}_{T_c}\right) \times 2\pi\, r_i^3 \times D(r_i) \times \left({}^{(R_2-R_1)}/{}_{N}\right)}{\int_{R_1}^{R_2} 2\pi D(r) r^3\, dr}. \qquad (1)$$

   where we have broken the cavity into N = 350 radial shells, each with radius r_{i}, ranging from R_1 = 2 mm to R_2 = 5.5 mm.

4. We select a trial value of $D/\xi_\Delta\left(P, \frac{T_{\mathrm{BA}}}{T_{\mathrm{c}}}\right) = \theta$. For any value of ${}^{T}/{}_{T_{\mathrm{c}}}$, we determine if a particular element r_i in S-1 has a value of $D(r_i)/\xi_\Delta(T/T_c) \geq \theta$. If the value is $\geq \theta$, then the element is taken to be in the B phase, if the value is $< \theta$, then it is taken to be in the A phase. We compute $\frac{\rho_{\mathrm{s}}^{\mathrm{Fit}}}{\rho}\left(\theta, {}^{T}/{}_{T_{\mathrm{c}}}\right)$ for a given value of $\theta, {}^{T}/{}_{T_{\mathrm{c}}}$.
5. We step ${}^{T}/{}_{T_{\mathrm{c}}}$ through the region of interest.
6. At the end of steps 1-5 we can generate $\frac{\rho_{\mathrm{s}}^{\mathrm{Fit}}}{\rho}\left(\theta, {}^{T}/{}_{T_{\mathrm{c}}}\right)$
7. The process is repeated for different values of $D/\xi_\Delta = \theta$

8. For a particular value of $D/\xi_\Delta = \theta$, we determine $T_{\mathrm{BA}}^{\mathrm{upper}}/T_\mathrm{c}$, $T_{\mathrm{BA}}^{\mathrm{lower}}/T_\mathrm{c}$. In particular, $T_{\mathrm{BA}}^{\mathrm{upper}}/T_\mathrm{c}$ is very sensitive to small changes in our choice for θ. However, $T_{\mathrm{BA}}^{\mathrm{upper}}/T_\mathrm{c}$ and its variance can be directly determined from the data as the upper location at which superfluid fraction data on cooling and warming join. We compare the fit to measured values and select the best fit. This establishes best fit values for D/ξ_Δ , $T_{\mathrm{BA}}^{\mathrm{upper}}/T_\mathrm{c}$, $T_{\mathrm{BA}}^{\mathrm{lower}}/T_\mathrm{c}$. Variances for the best fit for D/ξ_Δ are gauged when the fits clearly deviate from data. Values for $T_{\mathrm{BA}}^{\mathrm{lower}}/T_\mathrm{c}$ and their variance follow from the best fit and variance of D/ξ_Δ.
9. The process is repeated for maximum deflection values of 30 and 32 nm.bar^{-1}. Variation of these values affect the errors of $T_{\mathrm{BA}}^{\mathrm{lower}}/T_\mathrm{c}$. These are added quadratically to the error found in step 8. We also include the effect of a ±50 mbar uncertainty in pressure on the estimated deflection.
10. We used the observed values of $\mathrm{T}_{\mathrm{BA}}^{\mathrm{upper}}/T_\mathrm{c}$ and observed value of $T_{\mathrm{AB}}/T_\mathrm{c}$ for each cooling-warming pair of available temperature sweeps and calculated $\delta T/T_\mathrm{c} = T_{\mathrm{BA}}^{\mathrm{upper}}/T_\mathrm{c} - T_{\mathrm{AB}}/T_\mathrm{c}$ for each run. We then computed the mean supercooling $\delta T/T_\mathrm{c}$ and its standard error.

Supplementary Note 2: Location of the site of Nucleation of the B phase from the A phase.

The B phase nucleation is expected to occur at some location in the most bowed region of the cell. Here the B phase is thermodynamically the most stable phase. This is directly supported by the data at 2.5, 3.6 and 5.6 bar, by comparing the evolution of superfluid density through the AB transition region, with the BA transition, modeled as discussed in detail in Supplementary Note 1. In this model the motion of the interface (growth of the A phase) is governed by a transition from B to A phase at some fixed, pressure dependent value of D/ξ_Δ. We observe the agreement between the superfluid density measured below $T_{\mathrm{AB}}/T_{\mathrm{c}}$, after nucleation of the B phase in this bowed region of the cell with that observed while warming as seen in Fig. 3 and Fig. 5 of the main text.

This picture is also supported by prior NMR studies of the AB transition.[3] Nucleation of a bubble of the B phase towards the periphery in the more confined regions should lead to its collapse as both the surface energy and volume energies preclude growth of the bubble. The precise location of the site of the B phase nucleation event is not determined experimentally in this work, but might be resolved by NMR. Theoretical determination of the optimal location, within the resonant tunneling model, would be of great interest.

At those pressures where the supercooling exceeds the width of the transition region (0.1, 1.4 bar), we cannot rule out processes preempting intrinsic nucleation, such as the propagation of the B-phase into the slab from the fill line; therefore the supercooling observed at these low pressures provides a lower bound on the threshold of the intrinsic nucleation of the B phase under confinement.

Supplementary Note 3: Brief summary of the Resonant Tunneling Model.

It is well established that, in bulk superfluid ^{3}He, the conventional homogeneous nucleation model dramatically fails to account for the nucleation of the B phase from the A phase.[4,5] The predicted lifetime of the supercooled A phase, using known free energy and interfacial surface energy parameters exceeds, by a large factor, the lifetime of the universe.[6] Heterogeneous nucleation by impurities is precluded by the fact that ^{3}He is impurity-free at low temperatures.

This paradox has been addressed by theoretical models, which invoke extrinsic nucleation.[4,5,7,8,9] While there is support from experiments for such mechanisms, there is also evidence that B-phase nucleation in bulk takes place at a specific temperature,[10,11] which can be interpreted as pointing to some intrinsic nucleation mechanism.

In outline, the resonant tunneling model[12] is based on the notion that quantum tunneling between a supercooled metastable A phase and a stable B phase will be enhanced by an intermediate free energy minimum (false vacuum). The order parameter of superfluid ^{3}He is described by a 3×3 matrix of complex numbers; a manifold of 18 dimensions. The elaborate landscape of the free energy over this manifold opens the possibility of several potential resonant tunneling pathways, in some of which the tunneling rate may be substantially enhanced in a particular region of parameter space: temperature, pressure, magnetic field, rotation. Resonant tunneling therefore provides a model for intrinsic nucleation, under the right specific conditions.

In our nanoscale cavity geometry, the putative stripe phase, naturally provides the intermediate "false vacuum" required by the resonant tunneling model. As discussed in the main manuscript, the stripe phase is a spatially modulated phase of B-phase domains. The **q**-vector depends on confinement and temperature (in a geometry of constant thickness) and should also therefore have in-plane spatial variation due to bowing. We argue that these virtual or real

"striped phase variants" function as intermediaries promoting resonant tunneling of the system from A to B phase, under optimum temperature conditions. In the scenario in which the stripe phase is not stable, we expect the relevant temperature range to be a narrow region near the equilibrium AB transition line, close to that for which the stripe phase would be stable for slightly modified strong coupling parameters. Theoretical predictions of tunneling rates are desirable.

In the present experiment we were constrained to traverse the A-B region at a slow rate (to observe the A to B transition). Thus the transition had time to be reliably seeded at this optimal temperature-pressure. Further studies of the stochastics of B phase nucleation, and possible supercooling of A-phase for different rapid cooling rates to low temperatures, should test this nucleation model. Observation of the A-B transition over a wider pressure range, to adjust the energetics of the stripe phase in the multidimensional energy landscape, and potentially suppress resonant tunneling are also envisaged.

Supplementary References

[1] Wiman, J.J. and Sauls, J.A. Strong-coupling and the stripe phase of ^{3}He. *J. Low Temp. Phys.* **184** 1054-1070 (2016).

[2] Sauls, J.A and Serene, J.W. Potential scattering models for the quasiparticle interactions in liquid ^{3}He. *Phys. Rev. B*, **24**, 183-189, (1981).

[3] Levitin, L.V., Bennett, R.G., Casey, A., Cowan, B., Saunders, J., Drung, D., Schurig, Th., and Parpia, J.M. Phase diagram of the topological superfluid 3He confined in a nanoscale slab geometry. *Science* **340**, 841-844, (2013).

[4] Leggett, A.J. and Yip, S.K. Nucleation and growth of 3He-B in the supercooled A-phase. In *Helium Three* (eds Pitaevskii, L. P. and Halperin, W. P.) Ch. 8 (North Holland, 1990).

[5] Leggett, A.J. The ^{3}He A-B interface. *J. Low Temp. Phys.* **87** 571-593, (1992).

[6] Leggett, A.J. Nucleation of 3He-*B* from the *A* phase: A cosmic-ray effect? *Phys. Rev. Lett.* **53** 1096-1099 (1984).

[7] Leggett, A.J. High-energy low-temperature physics: Production of phase transitions and topological defects by energetic particles in superfluid 3He. *J. Low Temp. Phys.,* **126** 775-804 (2002).

[8] Bunkov, Yu. The multiuniverse transition in superfluid 3He. *J. Phys: Condens. Matter* **25** 404205 (2013).

[9] Bunkov, Yu. M., Timofeevskaya, O.D. “Cosmological” scenario for A− B phase transition in superfluid 3He. *Phys. Rev. Lett*. **80**, 4927-4930 (1998).

[10] Hakonen, P.J., Krusius, M., Salomaa, M.M., and Simola, J.T. Comment on “Nucleation of 3He-B from the A phase: A cosmic ray effect?” *Phys. Rev. Lett.* **54** 245 (1985).

[11] Leggett, A.J., “Leggett responds” *Phys. Rev. Lett.* **54** 246 (1985).

[12] Tye, S.-H.H., and Wohns, D. Resonant tunneling in superfluid He. *Phys. Rev. B* **84**, 184518 (2011).
